# Polyclonal *Burkholderia cepacia* complex outbreak caused by contaminated chlorhexidine gluconate solution

**DOI:** 10.1017/ash.2023.358

**Published:** 2023-09-29

**Authors:** Christel Valdez, Cybele Abad, Karl Evans Henson, Mark Carascal, Raul Destura

## Abstract

**Background:** *Burkholderia cepacia* complex is an opportunistic environmental pathogen that has been linked to nosocomial outbreaks. We describe an outbreak of bacteremia caused by *Burkholderia cenocepacia* from a contaminated chlorhexidine gluconate solution. **Methods:** The hospital infection control team carried out an outbreak investigation on February 21, 2021, when 3 adult hemodialysis patients developed *B. cenocepacia* bacteremia. Patient demographics and clinical profile were reviewed retrospectively. Potential sources of infection were identified, and environmental screening was performed in several units. Processes of catheter care in the hemodialysis unit were reviewed. Water samples from the hemodialysis unit, and samples of solutions used in patient care were sent for culture. Isolates from patients and from environmental samples were sent for 16S rRNA gene sequencing to determine genetic relatedness. **Results:** In total, 16 patients, 8 of whom were male, developed *B. cenocepacia* bacteremia during the investigated period. The median age was 68 years (range, 19–83), and 15 of 16 had at least 1 comorbidity. All patients used a central venous catheter (CVC) for hemodialysis, and 11 (70%) of these 16 were temporary. Chlorhexidine gluconate solution was routinely used as part of CVC care and 1 bottle was shared among 4 hemodialysis stations. On suspicion of contamination, all identified chlorhexidine bottles were recalled on February 26, 2021, and random samples from 15 opened and 19 unopened bottles were sent for culture from the following units: hemodialysis (n = 2), ICU (n = 14), wards (n = 6), and 4 each from transplant surgery, and delivery suites. O0f 34 sampled bottles, 17 grew *B. cenocepacia*: 8 opened and 9 unopened bottles. The Bayesian inference tree (Fig. 1) supports the hypothesis that patient samples and the samples from the chlorhexidine solutions were most probably related to each other based on the 16S rRNA sequences. However, the individual identities of the specific sample sequences could not be determined using the analyzed region of the gene, possibly due to low quality of the sequences received. No new cases of *B. cenocepacia* were identified after recall of the chlorhexidine solution, and the outbreak was deemed resolved on March 24, 2021. **Conclusions:** Medical solutions routinely used in patient care can cause outbreaks and should be suspected as a potential source of infection by infection control teams.

**Figure:**
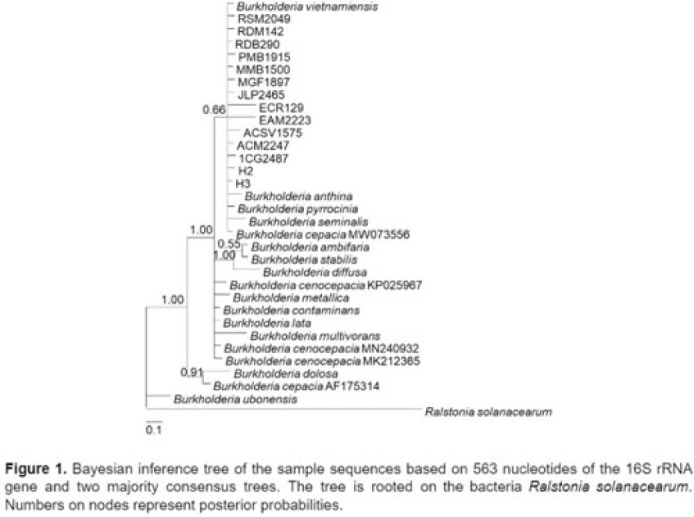

**Disclosures:** None

